# Association between higher eating frequency and lower odds of low muscle mass in Koreans

**DOI:** 10.3389/fmed.2025.1663242

**Published:** 2026-01-20

**Authors:** Mose Chun, Sae Rom Lee

**Affiliations:** 1 Department of Emergency Medicine, Pusan National University Yangsan Hospital, Yangsan, Republic of Korea; 2 Research Institute for Convergence of Biomedical Science and Technology, Pusan National University Yangsan Hospital, Yangsan, Republic of Korea; 3 Department of Family Medicine, Pusan National University Yangsan Hospital, Yangsan, Republic of Korea

**Keywords:** eating frequency, KoGES cohort, meal pattern, middle-aged and older adults, muscle mass, nutrition, sarcopenia

## Abstract

**Background:**

Sarcopenia, an age-related decline in muscle mass and function, is a growing public health concern. While protein intake and exercise are known management strategies, the independent role of meal frequency remains under-explored. This study investigated the association between eating frequency and low muscle mass in middle-aged and older Koreans.

**Methods:**

Using cross-sectional data from the KoGES cohort (*n* = 6,427), we categorized participants into low (1–2 meals/day) and high (3–5 meals/day) eating frequency groups. Low muscle mass was defined as a Muscle Mass Index (MMI, kg/m^2^) below the sex-specific 20th percentile. We employed binary logistic regression, adjusting for comprehensive confounders including age, sex, BMI, total protein intake, and physical activity.

**Results:**

Our findings revealed that a higher eating frequency (three or more meals per day) was independently associated with a significantly low odds ratio of low muscle mass (OR = 0.685, 95% CI: 0.566–0.828, *p* < 0.001), even after adjusting for total protein intake. This suggests that regular meal patterns, beyond total nutrient quantity, may be important for muscle maintenance. Potential mechanisms include optimized insulin secretion and avoidance of chronic energy deficits.

**Conclusion:**

Despite its cross-sectional design, this study offers novel insights from a large cohort, highlighting meal frequency as a potentially important, yet over-looked, nutritional factor associated with lower odds of low muscle mass. Our results support incorporating regular meal frequency recommendations into strategies for healthy aging.

## Introduction

Sarcopenia, a progressive age-related decline in muscle mass, strength, and function, significantly contributes to morbidity and mortality in the elderly population (1). The growing geriatric demographic has spurred extensive research into sarcopenia, revealing its strong association with increased frailty, heightened vulnerability to physical functional decline, systemic impairments, and various diseases (2). In adults, sarcopenia has been consistently linked to an elevated incidence of falls and fractures, Type 2 Diabetes Mellitus, cardiovascular disease, and metabolic syndrome (3–6). Furthermore, in older adults, sarcopenia impairs physical function, compromising independent living (7), and in hospitalized patients, it is associated with prolonged hospital stays, increased complications (8), higher readmission rates, and elevated mortality risk (9).

Current research endeavors to prevent and treat sarcopenia involve various compounds such as selective androgen receptor modulators, growth hormone and growth hormone secretagogues, insulin-like growth factor, and creatine (10–13). However, none of these agents have yet received official approval from the Food and Drug Administration (FDA) and remain in research phases or are limited to specific patient populations. Thus, non-pharmacological interventions, particularly lifestyle and nutritional strategies, are the cornerstone of sarcopenia management, as highlighted by the 2019 Asian Working Group for Sarcopenia (AWGS) consensus (14). Nutritional strategies, in particular, are considered a pivotal factor. Previous studies have demonstrated a significant association between lower daily total protein intake and sarcopenia (15–17). Beyond the total quantity, studies have advanced our understanding of the importance of eating patterns. For instance, research suggests that the distribution of protein across meals influences net protein balance, with an even protein distribution aiding in sarcopenia prevention (18). The findings from these studies on protein distribution inherently highlight the importance of the *timing* and *frequency* of nutrient intake in regulating muscle protein synthesis (MPS) (19). However, the effect of overall meal frequency, independent of total protein intake, is an under-explored area. This distinction is critical because high meal frequency may inherently improve protein partitioning or overcome age-related anabolic resistance (20), suggesting that the pattern of nutrient delivery itself may be key, regardless of calculated daily protein quantity. However, due to data limitations in the KoGES cohort, this study defines the outcome as low muscle mass using a sex-specific MMI percentile, rather than the full sarcopenia definition (including strength and function) or the absolute ASM/height^2^ cut-points recommended by AWGS. This study therefore aims to investigate the association between eating frequency and low muscle mass using the KoGES cohort database in a middle-aged and older Korean population, positioning our work as hypothesis-generating for future interventional studies.

## Materials and methods

### Study population

This study employed a retrospective cohort design utilizing the KoGES (Korean Genome and Epidemiology Study) dataset, collected by the Korea Disease Control and Prevention Agency’s National Institute of Health. The KoGES dataset comprises 10,030 male and female participants aged 40–69 years residing in urban (Ansan) and rural (Anseong) areas. This ongoing study began its first wave in 2001, with follow-up surveys conducted biennially, and the 10th wave completed in 2022 (21). For the purpose of this analysis, which focused on the association between baseline eating frequency and sarcopenia, we employed a cross-sectional analysis utilizing only the 2001 baseline survey data, as the specific questions regarding eating frequency were exclusively included in this first wave. The final sample used for analysis was derived from the 2001 KoGES baseline dataset. The KoGES dataset includes self-reported demographic and socioeconomic characteristics, medical history, smoking and alcohol consumption habits, dietary intake, and physical activity levels, along with comprehensive physical measurements, urine, and blood test results. Dietary information was collected using a semi-food frequency questionnaire (FFQ) developed by KoGES. This 103-item semi-FFQ was specifically designed and validated for the Korean population, demonstrating acceptable validity and reproducibility when compared with 3-day food records and biochemical indicators in prior studies (22). As questions regarding eating frequency were exclusively included in the first baseline survey in 2001, the current study employed a cross-sectional analysis utilizing the 2001 KoGES dataset. From the initial 10,300 participants, approximately 3,873 participants were excluded due to missing data. Specifically, 607 participants were excluded for missing covariate data (education, marital, alcohol, smoking status), 3,013 were further excluded for missing BIA record data, and additional 253 were excluded for missing meals record data. This resulted in 6,427 participants included in the final analysis, who were then categorized into the low meal frequency group (*n* = 814) and the higher meal frequency group (*n* = 5,613). This was handled using a Complete Case Analysis (CCA), based on the assumption that data were missing completely at random (MCAR) or missing at random (MAR), a standard approach for this dataset.

### Muscle mass index

While current consensus guidelines for sarcopenia (AWGS) primarily recommend using the Appendicular Skeletal Muscle Mass Index (ASMI) and require assessments of muscle strength and/or physical performance, the KoGES 2001 baseline data utilized in this cross-sectional analysis unfortunately only provided Total Muscle Mass. Crucially, data for appendicular muscle mass, muscle strength (e.g., handgrip strength), and physical performance (e.g., gait speed) were not included in this dataset. Therefore, it was epidemiologically infeasible to define sarcopenia using the full consensus criteria. As a robust surrogate measure for low muscle mass in this population, muscle mass was assessed using multi-frequency bioelectrical impedance analysis (Inbody 3.0, Biospace, Seoul, Korea). The BIA measurement was conducted under standardized conditions, typically after an overnight fast and in a supine position, following the manufacturer’s protocol. The Muscle Mass Index (MMI) was calculated by dividing total muscle mass by the square of height (kg/m^2^), serving as a measure of muscle mass relative to stature. A reduction in muscle mass was defined by an MMI value falling below the 20th percentile, separately for males and females, categorizing participants into the muscle mass reduction group.

### Eating frequency and covariates

Eating frequency in 2001 was assessed via a self-administered questionnaire. Participants were asked, ‘How many meals do you usually consume per day?’ with an open-ended response format. The question specifically asked for the number of main meals consumed, and did not differentiate between breakfast, lunch, or dinner. Crucially, the definition of “meal” used in the questionnaire explicitly excluded snacks. For this study, participants reporting 1–2 regular meals per day were categorized into the low eating frequency group, while those reporting 3–5 meals per day were classified into the high eating frequency group. This dichotomization was chosen to reflect a clear public health distinction between irregular (less than daily routine) and regular meal patterns. Covariates included age, sex, smoking status, alcohol consumption status, daily physical activity, and daily protein intake, all obtained through self-reported questionnaires. Smoking Status was categorized based on the self-reported response to the question, ‘Have you ever smoked?’ participants who responded ‘No’ were classified as Non-smokers. Those who responded ‘Yes’ were further categorized: ex-smokers if they indicated past smoking, and current smokers if they indicated smoker status. Alcohol Consumption Status was categorized based on the response to the question, ‘Do you usually not drink alcohol, or have you never consumed alcohol (e.g., due to religious reasons)?’ Participants who responded ‘Yes’ to this question were classified as non-drinkers. Those who responded ‘No’ were further categorized: Past drinkers if they indicated previous consumption but had quit, and current drinkers if they reported current alcohol consumption. Educational Attainment was categorized into three groups based on the response to the question, ‘What is the highest level of education you have completed?’ The three defined categories were: Middle school graduate or lower, College graduate (or equivalent), and Postgraduate or higher. Marital status was divided into single, married, widowed, or separated/divorced. Household Monthly Income was categorized into four groups based on the self-reported response to the question, ‘What is the approximate average monthly income of your household?’ The four defined categories were: ≤ 1.5 million Korean Won (KRW), > 1.5 million KRW and ≤ 3 million KRW, > 3 million KRW and ≤6 million KRW, > 6 million KRW. Daily protein intake was measured using the semi-food frequency questionnaire (FFQ). The total daily protein intake (g/day) was calculated by multiplying the frequency of consumption for each food item by its nutrient content, using the KoGES specific nutritional database derived from the Korean Food Composition Table. Physical Activity was quantified using Metabolic Equivalent of Task (MET) values. The total physical activity was calculated as the sum of the product of the MET score for each self-reported activity (light, moderate, and vigorous) multiplied by its duration (hours/week). This final value was reported in the study as MET-hours/week. Body Mass Index (BMI) (kg/m^2^) was calculated as weight divided by the square of height. Although MMI uses height^2^ in its denominator, BMI was included as a covariate to adjust for potential residual confounding by overall adiposity, which is a known predictor of both eating behavior and muscle mass status.

### Statistical analyses

**Figure 1:**
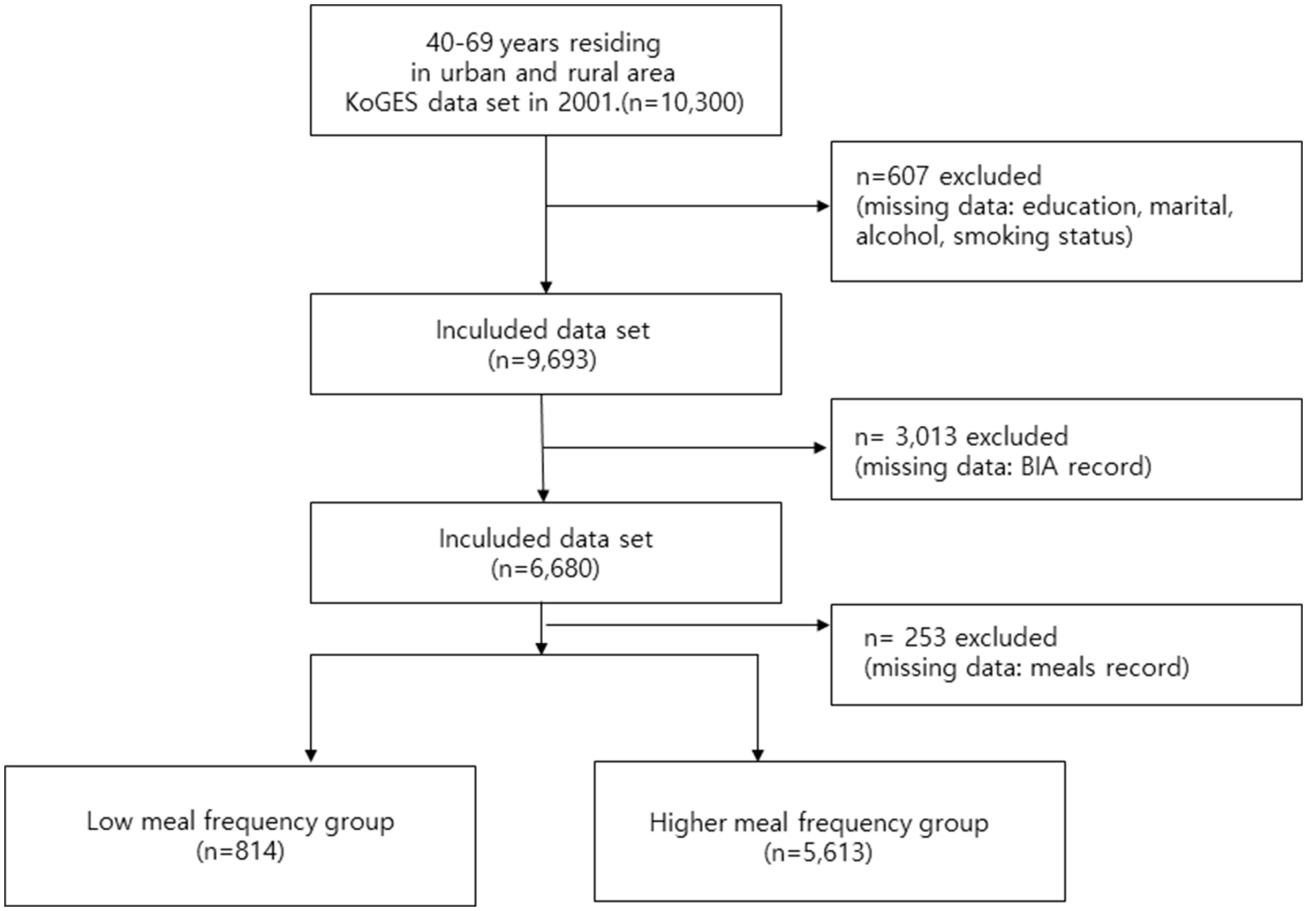
Flow diagram of participant selection for the study.

Baseline characteristics of continuous variables were presented as means ± standard deviations, while categorical variables were expressed as numbers and percentages. To assess significant differences between variables, the *t*-test was employed for continuous variables, and the chi-squared test was used for categorical variables. The Odds Ratio (OR) with 95% confidence intervals (CI) for the muscle mass reduction group (defined as MMI below the 20th percentile) was determined using binary logistic regression analysis. In the unadjusted model of the binary logistic regression, the ORs for the MMI below the 20th percentile were assessed based on eating frequency. Model 1 adjusted for age, sex, BMI, protein intake, and physical activity. Model 2 further adjusted for education level, marital status, income, smoking status, and alcohol consumption status, in addition to the variables included in Model 1. To address concerns regarding the potential collinearity between the Muscle Mass Index (MMI) and Body Mass Index (BMI), given that both utilize height squared (m^2^) in the denominator, a sensitivity analysis was performed. This analysis repeated the full binary logistic regression (Models 1 and 2) with BMI excluded as a covariate (Supplementary Table S1). Furthermore, to explore the association of low muscle mass across different meal consumption levels, a second sensitivity analysis was conducted using an ordinal (multi-category) eating-frequency model. This model categorized meal frequency into four groups (1 meal/day as reference, 2 meals/day, 3 meals/day, and 4 meals/day) and also excluded BMI from the adjustment models (Supplementary Table S2). All statistical analyses were performed using SPSS software (version 28.0; IBM Corp., Armonk, NY, USA). The level of statistical significance (alpha threshold) was set at *p* < 0.05 (Figure 1).

## Results

### Baseline characteristics of participants

Table 1 presents the baseline characteristics of participants stratified by meal frequency group: low meal frequency (1–2 meals/day) and high meal frequency (3–5 meals/day). A total of 814 participants (12.7%) were in the low meal frequency group, while 5,613 participants (87.3%) were in the higher meal frequency group. The unadjusted prevalence of low muscle mass was 23.8% (*n* = 194) in the low meal frequency group and 19.4% (*n* = 1,089) in the high meal frequency group. Significant differences were observed between the two groups for several baseline characteristics. Participants in the high meal frequency group were significantly older (51.9 ± 8.8 years vs. 50.3 ± 8.6 years, *p* < 0.001). Regarding education, the high meal frequency group had a significantly greater proportion of individuals with middle school graduate or lower (55.2% vs. 43.6%, *p* < 0.001) and a lower proportion with Postgraduate degree or higher (9.6% vs. 15.4%). Significant differences were also found in income levels (*p* < 0.001), with the high meal frequency group having a larger proportion in the ≤ 1.5 million KRW/month category (50.1% vs. 38.7%). Smoking status also differed significantly (*p* < 0.001), where the high meal frequency group had a lower proportion of non-smokers (50.0% vs. 57.0%) and a higher proportion of ex-smokers (20.3% vs. 8.4%) and current smokers (29.7% vs. 12.8%). Furthermore, the high meal frequency group exhibited significantly higher physical activity levels as measured by MET (8996.9 ± 5310.4 vs. 7415.9 ± 4039.3, *p* < 0.001) and higher daily protein intake (68.1 ± 29.1 g vs. 63.4 ± 26.0 g, *p* < 0.001). Conversely, the high meal frequency group had a significantly lower BMI (25.4 ± 2.9 kg/m^2^ vs. 26.1 ± 3.0 kg/m^2^, *p* < 0.001).

**Table 1:** Baseline characteristics of the participants.

Characteristics	Low meal frequency group (*n* = 814)	Higher meal frequency group (*n* = 5,613)	*p*-value
Age (years)	50.3 ± 8.6	51.9 ± 8.8	< 0.001
Men, *n* (%)	404 (48.6)	2,685 (47.8)	0.338
Education			
≤ Middle school graduation	355 (43.6)	3,100 (55.2)	<0.001
≤ University graduate	334 (41.0)	1976 (35.2)	
Postgraduate degree or higher	125 (15.4)	537 (9.6)	
Marry status			
Never married	10 (1.2)	86 (1.5)	0.473
Married	745 (91.5)	5,039 (89.8)	
Widowed	45 (5.5)	381 (6.8)	
Separated or divorced	14 (1.7)	107 (1.9)	
Income			
≤ 1.5 million KRW	315 (38.7)	2,813 (50.1)	< 0.001
≤ 3 million KRW	306 (37.6)	1807 (32.2)	
≤ 6 million KRW	167 (20.5)	873 (15.6)	
> 6 million KRW	26 (3.2)	120 (2.1)	
Drinking			
Non-drinker	338 (41.5)	2,220 (39.5)	0.093
Ex-drinker	43 (5.3)	409 (7.3)	
Current drinker	433 (53.2)	2,984 (53.2)	
Smoking status			
Non-smoker	464 (57.0)	2,809 (50.0)	<0.001
Ex-smoker	105 (8.4)	1,139 (20.3)	
Current-smoker	245 (12.8)	1,665 (29.7)	
METs (kcal/h)	7415.9 ± 4039.3	8996.9 ± 5310.4	< 0.001
Protein intake (g/day)	63.4 ± 26.0	68.1 ± 29.1	< 0.001
BMI (kg/m^2^)	26.1 ± 3.0	25.4 ± 2.9	< 0.001
MMI (kg/m^2^)	17.5 ± 1.4	17.6 ± 1.4	0.053

BMI, body mass index; KRW, Korean Won; METs, metabolic equivalents; MMI, muscle mass index. Data are presented as number (with percentage) of patients, mean ± standard deviation for continuous data. *p*-value was obtained by student’s test or by chi-square test.

### Association of meal frequency with low muscle mass

Table 2 presents the Odds Ratios (ORs) and 95% Confidence Intervals (CIs) for low muscle mass (defined as MMI below the 20th percentile) according to meal frequency group, with the low meal frequency group (low frequency meal) serving as the reference. In the unadjusted model, individuals in the high meal frequency group (high frequency meal) showed a significantly lower odds of low muscle mass (OR = 0.775, 95% CI: 0.675–0.888, *p* < 0.001) compared to the low meal frequency group. After adjusting for age, sex, BMI, total protein intake, and physical activity (Model 1), the association remained robust. The high meal frequency group had a significantly lower odds of low muscle mass (OR = 0.628, 95% CI: 0.532–0.742, *p* < 0.001). Further comprehensive adjustment in Model 2 (including age, education, marital status, income, drinking status, smoking status, physical activity, total protein intake, and BMI) did not attenuate this significant association. The high meal frequency group continued to demonstrate a significantly lower odds of low muscle mass (OR = 0.685, 95% CI: 0.566–0.828, *p* < 0.001). To assess the robustness and potential confounding of the primary findings, we conducted two sensitivity analyses. First, when BMI was excluded from the fully adjusted models (Models 1 and 2) to assess the primary association without potential over-adjustment, the inverse relationship between high meal frequency (3–5 meals/day) and low muscle mass was attenuated and became statistically non-significant (Model 2 OR = 0.853, 95% CI: 0.713–1.021, *p* = 0.083) (Supplementary Table S1). Second, the ordinal (multi-category) model examining the odds ratio of low muscle mass for 2, 3, and 4 meals per day (referenced against 1 meal per day) after excluding BMI did not reveal a statistically significant association or a clear dose–response trend across the eating frequency categories in any adjusted model (Model 2 *p* = 0.750) (Supplementary Table S2).

**Table 2:** Odd ratio and 95% confidence intervals for low muscle mass according meal frequency.

Models	Low frequency meal (reference)	High frequency meal OR (95% CI)	*p*-value
Unadjusted model	1	0.775 (0.675–0.888)	<0.001
Model 1	1	0.628 (0.532–0.742)	< 0.001
Model 2	1	0.685 (0.566–0.828)	<0.001

CI, confidence interval; OR, odds ratio. Data are expressed as odds ratio (95%, confidence interval). Model 1 adjusted for age, sex, BMI, protein intake, physical activity. Model 2 adjusted for Model1 + education level, marital status, income, smoking status, and alcohol consumption status.

## Discussion

Our study found that consuming three or more meals per day, which is indicative of higher eating frequency, was associated with lower odds of low muscle mass. This finding is significant given the current understanding of sarcopenia prevention. Sarcopenia is a growing public health concern, and optimal nutritional strategies are crucial for its prevention and management. Extensive research has consistently highlighted the importance of adequate protein intake in middle-aged and older adults. For instance, a systematic review and meta-analysis by Bauer et al. concluded that higher protein intake is associated with greater muscle mass and strength in older adults, suggesting a crucial role in preventing sarcopenia (23). Similarly, Phillips et al., while primarily focusing on athletes, broadly discussed the importance of sufficient protein to counteract muscle protein breakdown, a principle highly relevant to age-related muscle loss (24). These studies underscore the fundamental need for older adults to consume sufficient protein to mitigate sarcopenia. Beyond total protein quantity, the frequency and distribution of protein intake throughout the day have also gained attention. Research suggests that spreading protein intake evenly across meals may be more beneficial for stimulating muscle protein synthesis (MPS) than consuming large amounts in one or two meals. For example, Mamerow et al. (25) demonstrated that consuming protein more evenly throughout the day, rather than primarily at dinner, led to higher 24-h muscle protein synthesis rates. This supports the idea that consistent protein availability is key for muscle maintenance. Another relevant study by Areta et al. (26), while focusing on exercise recovery, provided evidence that distributing protein intake in moderate doses every few hours is more effective for MPS than fewer, larger doses. Although these studies are not exclusively focused on sarcopenia, their findings on MPS mechanisms are directly applicable to preventing age-related muscle loss. Our study uniquely contributes to this body of knowledge by suggesting that even after adjusting for total protein intake, higher eating frequency (3 or more meals per day) may be independently associated with a lower risk of reduced muscle mass. This is a novel finding, as previous research directly linking meal frequency, independent of total protein intake, to sarcopenia prevention has been scarce. While the precise mechanisms underlying this association are not yet definitively established, several possibilities can be considered, directly liking higher eating frequency to favorable metabolic states. Low frequent meals may lead to prolonged periods without nutrient intake, potentially decreasing insulin secretion. Reduced insulin levels can tip the balance toward a more catabolic state, promoting muscle protein breakdown (proteolysis) over synthesis (27). Conversely, higher meal frequency may facilitate insulin optimization, leading to a more consistent anabolic environment necessary for MPS (25). Specifically, distributing protein into discrete, adequate per-meal doses across the day is critical for overcoming age related anabolic resistance and maximizing the postprandial MPS response. A higher meal frequency pattern is hypothesized to naturally support this optimal protein distribution. Furthermore, if a lower meal frequency leads to a reduction in total energy intake, it could result in a broader deficiency of not only protein but also other essential macronutrients and micronutrients necessary for muscle metabolism and overall tissue maintenance (28). A higher eating frequency, by preventing these chronic energy and nutrient deficits, is hypothesized to sustain energy balance and mitigate catabolism, thereby increasing the effective availability of nutrients for muscle anabolism. We must acknowledge the potential for reverse causation inherent in our cross-sectional design. For instance, individuals experiencing subclinical frailty, illness, or anorexia of aging (a known factor in sarcopenia progression) might naturally reduce their appetite and, consequently, their meal frequency (29). This cycle (illness, low appetite, low meal frequency, low muscle mass) suggests that low muscle mass could be the consequence, rather than the precursor, of lower meal frequency. Furthermore, despite meticulous adjustment, the possibility of residual confounding from unmeasured factors (like specific comorbidity burden or detailed frailty status) cannot be fully excluded.

Despite its significant findings, our study has several limitations that warrant consideration. First, as a cross-sectional study, it can only identify associations and cannot establish causality between eating frequency and low muscle mass. The observed relationship could be bidirectional; for instance, individuals with low muscle mass might eat less frequently due to reduced appetite or functional limitations. Second, eating frequency was assessed via self-reported questionnaires, which are subjective and prone to recall bias, potentially affecting the accuracy of the dietary assessment. Third, a significant limitation stems from the age of the data used. This analysis relied exclusively on the KoGES baseline survey from 2001. Korean dietary habits and food availability have changed substantially over the past two decades, meaning the reported eating frequency and dietary intake may not fully reflect current Korean nutritional patterns. Fourth, the lack of crucial confounding variables must be addressed. Although we meticulously adjusted for total protein intake, our data lacked specific information on total energy intake and the protein distribution across individual meals. The frequency effect could be confounded if higher eating frequency simply serves as a proxy for higher overall energy intake or better protein distribution. Therefore, we cannot definitively rule out that these unmeasured nutritional factors may influence the observed association between meal frequency and low muscle mass. Fifth, the use of Complete Case Analysis (CCA), while standard for this dataset, carries the risk of selection bias if the missing data pattern was not truly random. Crucially, our analytic sample excluded over 37% of the total baseline participants due to missing data in key variables. The considerable extent of this data exclusion warrants a cautious interpretation of the results and suggests a potential for selection bias, as the characteristics of the excluded participants may systematically differ from those included in the final analysis. Lastly, our adjustment for comorbidity was limited to self-reported status, and detailed comorbidity scores or frailty indexes were not available, potentially leaving residual confounding. Nevertheless, our study possesses several notable strengths. To our knowledge, this is one of the first large-scale studies to investigate the association between eating frequency and low muscle mass incidence in a Korean population. The use of the KoGES cohort database, a well-established and comprehensive dataset, allowed for the analysis of a large and representative sample, enhancing the generalizability of our findings within the Korean context. Furthermore, the study meticulously adjusted for a wide array of potential confounding variables, including demographic characteristics, socio-economic status, lifestyle factors (smoking, alcohol, physical activity), and crucial nutritional parameters (total protein intake, BMI). This rigorous adjustment strengthens the validity of the observed association between eating frequency and muscle mass outcomes, providing valuable insights into a relatively under-researched area of sarcopenia prevention.

These findings offer hypothesis-generating evidence that may inform nutritional research. While current guidelines emphasize adequate protein intake and regular exercise, our research suggests the need to consider meal frequency as an additional, practical nutritional factor associated with muscle health. Healthcare professionals should counsel middle-aged and older patients on the importance of regular meal patterns, recognizing this as preliminary evidence supporting cautious dietary counseling. This approach could be particularly beneficial for middle-aged and older patients who experience irregular eating patterns due to appetite loss or gastrointestinal issues. Need for Further Research: As this was a cross-sectional study, longitudinal prospective cohort studies and randomized controlled trials (RCTs) are essential to establish causality. Future research should directly evaluate the impact of changes in meal frequency on muscle mass and function, and meticulously analyze the quality and quantity of food consumed at each meal.

## Conclusion

Our study, utilizing data from the KoGES cohort, confirmed that a higher eating frequency (three or more meals per day) is independently associated with a lower odds of low muscle mass in middle-aged and older patients. This finding remained significant even after adjusting for total protein intake, thereby highlighting the importance of a higher meal frequency pattern, in addition to the quantity of food consumed, in nutritional strategies aimed at mitigating low muscle mass risk. While these findings are novel and provide important insights, our cross-sectional design prevents causal inference. Therefore, future research should prioritize longitudinal prospective cohort studies and randomized controlled trials to establish causality. Furthermore, future studies should employ standardized outcome definitions (e.g., AWGS/EWGSOP criteria including ASMI and function) and utilize more objective and validated instruments for dietary exposure measurement to confirm this association with greater precision.

## Data Availability

The raw data supporting the conclusions of this article will be made available by the authors, without undue reservation.
