# Supplementary material for: Association between higher eating frequency and lower odds of low muscle mass in Koreans
Source: Front Med (Lausanne). 2026 Jan 20;12:1663242. doi: 10.3389/fmed.2025.1663242 (PMC12864403; doi:10.3389/fmed.2025.1663242)
Supplement: Supplementary file 1 [file Table_1.docx]

**Table S1.** Odds Ratio and 95% confidence intervals for low muscle mass excluding BMI according by meal frequency.

|  | **Low frequency meal**  **(reference)** | **High frequency meal**  **OR (95% CI)** | **p-value** |
| --- | --- | --- | --- |
| Unadjusted model | 1 | 0.775 (0.675-0.888) | <0.001 |
| Model 1 | 1 | 0.853 (0.713-1.019) | 0.080 |
| Model 2 | 1 | 0.853 (0.713-1.021) | 0.083 |

CI = confidence interval; OR= odds ratio. Data are expressed as odds ratio (95%, confidence interval). Model 1 adjusted for age, sex, protein intake, physical activity. Model 2 adjusted for Model1 + education level, marital status, income, smoking status, and alcohol consumption status.
