# Supplementary material for: Association between higher eating frequency and lower odds of low muscle mass in Koreans
Source: Front Med (Lausanne). 2026 Jan 20;12:1663242. doi: 10.3389/fmed.2025.1663242 (PMC12864403; doi:10.3389/fmed.2025.1663242)
Supplement: Supplementary file 2 [file Table_2.docx]

**Table S2.** Odds Ratio and 95% confidence intervals for low muscle mass excluding BMI according by multi category meal frequency.

|  | **1 meal a day**  **(reference)** | **2 meal aday**  **(95% CI)** | **3 meal a day**  **(95% CI)** | **4 meal a day**  **(95% CI)** | **p-value** |
| --- | --- | --- | --- | --- | --- |
| Unadjusted model | 1 | 0.782 (0.205-2.976) | 0.650(0.172-2.455) | 0.842(0.168-4.227) | 0.147 |
| Model 1 | 1 | 0.822(0.221-3.437) | 0.597(0.150-2.387) | 0.632(0.116-3.457) | 0.822 |
| Model 2 | 1 | 0.781(0.171-3.562) | 0.563(0125-2.543) | 0.746(0.117-4.754) | 0.750 |

CI = confidence interval; OR= odds ratio. Data are expressed as odds ratio (95%, confidence interval). Model 1 adjusted for age, sex, protein intake, physical activity. Model 2 adjusted for Model1 + education level, marital status, income, smoking status, and alcohol consumption status.
